# Monthly simultaneous cannabis and alcohol use: effects on depression, anxiety, and stress in male and female college students

**DOI:** 10.1186/s42238-025-00347-y

**Published:** 2025-11-07

**Authors:** Eleftherios M. Hetelekides, Tabitha McMichael, Alexander J. Tyskiewicz, Mark A. Prince, Noah N. Emery, Bradley T. Conner, Hollis C. Karoly

**Affiliations:** 1 Department of Psychology, Colorado State University, Fort Collins, CO USA; 2 Department of Psychiatry and the Behavioral Sciences, Keck School of Medicine, Los Angeles, CA USA; 3 Department of Psychiatry, School of Medicine, University of Colorado Anschutz Medical Campus, Aurora, CO USA

**Keywords:** Polysubstance use, Frequency, Mental health, Sex differences, Young adults

## Abstract

**Background:**

Alcohol and cannabis are two of the most commonly used and co-used substances by young adults, and simultaneous cannabis and alcohol (SCA) use has been linked to increased risk for negative consequences, including poor mental health. College students represent an important group to study given their high prevalence of co-use and rising rates of depression, anxiety, and stress. Further, sex-related differences have been observed in polysubstance use, and research is needed on how SCA use may be differentially related to mental health outcomes in male and female college students.

**Methods:**

This cross-sectional survey study on *N* = 367 college students compared male and female sexes on the relationships between monthly SCA use (compared to less than monthly) and depression, anxiety, and stress. A multigroup path analysis was conducted to simultaneously regress mental health symptoms onto a dichotomously coded SCA group variable, while controlling for age, most recent cannabis use, and typical alcohol frequency, with sex as the grouping variable. A post-hoc Monte Carlo simulation was conducted to examine power and provide recommendations for sample sizes in future research.

**Results:**

Monthly SCA use significantly positively predicted depression in male (β = 0.322) and female (β = 0.296) college students, and the relation between SCA and depression was not different between sexes (*p* = 0.939). While anxiety (β = 0.323) and stress (β = 0.369) were significantly positively predicted by monthly SCA use in males, but not females, the relations between SCA and anxiety, as well as stress, were not significantly different between the sexes.

**Conclusions:**

Across sexes, more frequent SCA use is positively associated with depression symptoms in college students. Although SCA use was also linked to higher anxiety and stress among males but not females, these sex differences were not significant. Studies with greater statistical power are warranted to investigate potential sex differences.

## Background

From a nationally representative sample, 8.4% of U.S. adults aged 30 to 80 reported past-month co-use of alcohol and cannabis (Tucker et al. 2023). Among college students, the prevalence of non-disordered past-year co-use increased from approximately 1.8 million in 2002 to 2.6 million in 2018 (McCabe et al. 2021). Co-use is commonly categorized as either simultaneous (i.e., overlapping) co-use or concurrent (i.e., non-overlapping) co-use, based upon the temporal patterns of use. Simultaneous use of alcohol and cannabis (SCA) is defined as using both substances within the same timeframe, such that the intoxicating effects of each are experienced at the same time (Bravo et al. 2021; Cummings et al. 2019). SCA use occurs at the highest rate amongst young adults ages 19 to 22 (Terry-McElrath and Patrick 2018). Among college students who use both cannabis and alcohol, 79% reported past year SCA use with an average frequency of twice per month (White et al. 2019). Young adults often report engaging in SCA to experience overlapping effects, such as enhanced euphoria or relaxation (Jackson et al. 2020; Patrick et al. 2018, 2020).

SCA has been linked to a number of negative consequences when compared to non-overlapping alcohol and cannabis use, including increased consumption of both alcohol and cannabis (Cummings et al. 2019; Linden-Carmichael et al. 2019; Subbaraman and Kerr 2015) and greater odds of experiencing negative consequences including blackouts, impaired driving, physical injury, and acute psychological distress (Brière et al. 2011; Cummings et al. 2019; Haas et al. 2015; Jackson et al. 2020; Linden-Carmichael et al. 2019, 2020; White et al. 2019). High schoolers engaging in extreme binge drinking (i.e., more than 10 drinks in a row) and cannabis use are at elevated risk for engaging in SCA use, with frequency being a predicting factor (Patrick et al. 2017). Jackson and colleagues found that SCA use resulted in more severe negative consequences than non-overlapping use, even when accounting for frequency and quantity of use (Jackson et al. 2020), suggesting that the coinciding intoxication effects of SCA use may uniquely contribute to consequences (Subbaraman and Kerr 2015).

Mental health outcomes are significantly impacted by SCA use, particularly in adolescents and young adults. SCA use has been strongly correlated with increased depressive symptoms, lower grades, and a greater likelihood of psychosis and externalizing issues (Brière et al. 2011; Fleming et al. 2021; Patrick et al. 2022; Subbaraman and Kerr 2015; Thompson et al. 2021; Yurasek et al. 2017). Importantly, some longitudinal work has indicated that that SCA predicts subsequent increases in depressive symptoms, substance-related harms, externalizing problems, and psychosis risk across time (Fleming et al. 2021; Thompson et al. 2021). Furthermore, SCA use correlates with elevated incidence of mental health disorders and co-occurring substance use disorders (Subbaraman and Kerr 2015; Thompson et al. 2021; Yurasek et al. 2017). Prior work has categorized SCA frequency using a monthly cutoff (e.g., on the Alcohol and Cannabis Simultaneous Use Scale [ACSUS]; Kolp et al. 2024), and research indicates that reporting more frequent SCA is associated with elevated risk for substance-related consequences relative to less frequent co-use (Linden-Carmichael et al. 2019). Additionally, individuals endorsing monthly alcohol and/or cannabis use regularly demonstrate more pronounced mental health symptoms compared to individuals reporting less frequent use (Fleming et al. 2021; Karriker-Jaffe et al. 2018; Robinson et al. 2022; Sokolovsky et al. 2020; Subbaraman et al. 2019). These associations highlight the need to better understand the relationship between SCA use and mental health. This is particularly relevant for young adults and college students, who experience elevated rates of depression, anxiety, and stress (Beiter et al. 2015; Melchior et al. 2007; Ramón-Arbués et al. 2020) as well as high rates of SCA use (Terry-McElrath and Patrick 2018). Greater understanding of how SCA relates to mental health can offer clarity that may inform clinical interventions for young adults who co-use and are seeking mental health treatment. Further, because sex differences in internalizing disorders often begin to emerge in late childhood and adolescence (Hankin et al. 1998; Zahn-Waxler et al. 2008), it is also important to consider whether patterns of SCA use may interact with these developmental vulnerabilities.

Notably, prior research suggests that males and females may show different patterns of SCA use. Males may be more likely to engage in SCA and consume both alcohol and cannabis at higher quantities and frequencies than females (Erol and Karpyak 2015; Goodwin et al. 2022; Haberstick et al. 2014). On the other hand, females tend to experience similar subjective and objective effects when engaging in simultaneous use despite using significantly less cannabis than males (Wright et al. 2021). Understanding the differential associations of SCA with biological sex can offer valuable insights for clinical interventions aimed at reducing mental health symptoms in male and female college students who co-use these substances.

The present study aimed to explore whether SCA use at least once per month differently predicted scores on mental health variables (depression, anxiety, and stress) compared to individuals who engage in SCA less than once per month (including those who use alcohol and cannabis at non-overlapping times, but do not engage in SCA at all). Further, we aimed to examine whether relations between SCA use and depression, anxiety, and stress differed between males and females. Post-hoc Monte Carlo power simulations based on coefficient estimates from the current sample were examined to provide sample size recommendations for future studies and contextualize the present results.

## Methods

### Participants

The current study was a secondary data analysis of 367 participants from a University psychology student research pool. Participants were eligible for this study if they were enrolled as a college student at the University during the Fall 2022 semester, were between the ages of 18 and 25 years old and had not previously completed the study. Exclusion criteria included being under the age of 18 or over the age of 25, previously completing the study, and not being enrolled as a college student at the University during the Fall 2022 Semester. After gaining informed consent, eligible participants completed an online survey (which took approximately one hour to complete) inquiring about substance use behaviors and mental health. Participants answered demographic questions, followed by questions on alcohol and cannabis use. If a participant indicated that they ever used alcohol and cannabis, a question regarding SCA use frequency was populated. All participants were asked questions about mental health symptoms. As compensation for completing the survey, participants received class credit. The study was approved by the University Institutional Review Board. Participant data was excluded if they failed to provide complete responses to questions about alcohol and cannabis use or mental health symptoms or they did not indicate SCA use.

Participants (*N*_total_ = 367) were split into two groups: those who engaged in *monthly or greater SCA use* (henceforth referred to as the SCA + group; *N*_SCA+_ = 133) and those who engaged in *less than monthly SCA use* (henceforth referred to as the SCA- group; *N*_SCA−_ = 234). Frequency of participant SCA use within the SCA + group is described in Table 1. Basic demographic characteristics of both SCA + and SCA- groups can be found in Table 2.

**Table 1:** Frequency of SCA use within SCA + Group

Frequency of SCA Use	*N* (% Relative to SCA + Group Total) *N* = 133
1 Day/Month	26 (19.5)
2 Days/Month	26 (19.5)
3 Days/Month	25 (18.8)
1 Day/Week	32 (24.1)
2 Days/Week	19 (14.3)
3 Days/Week	4 (3.0)
4 Days/Week	0 (0)
5 Days/Week	0 (0)
6 Days/Week	1 (0.8)
7 Days/Week	0 (0)

Note. *SCA* Simultaneous Cannabis and Alcohol Use. SCA + indicates individuals reporting monthly simultaneous cannabis and alcohol use.

**Table 2:** Demographic characteristics of SCA + and SCA- groups

		SCA+ (At least monthly) *N* = 133	SCA- (Less than monthly) *N* = 234
Age (SD)		19.09 (1.50)	19.11 (1.44)
Sex (%)	Female	82 (61.7)	163 (69.7)
	Male	51 (38.3)	71 (30.3)
Gender (%)	Woman	66 (49.6)	134 (57.3)
	Man	48 (36.1)	61 (26.1)
	Androgynous	0 (0)	1 (0.4)
	Gender Fluid	1 (0.8)	0 (0)
	Gender Non-Binary	1 (0.8)	1 (0.4)
	Another	1 (0.8)	1 (0.4)
	Do Not Wish to Respond	15 (11.3)	36 (15.4)
Race (%)	American Indian or Alaska Native	2 (1.5)	2 (0.9)
	Asian	2 (1.5)	6 (2.6)
	Black or African American	1 (0.8)	3 (1.3)
	Native Hawaiian or Other pacific Islander	2 (1.5)	1 (0.4)
	White	119 (89.5)	196 (83.8)
	Do Not Wish to Respond	7 (5.3)	26 (11.1)
Ethnicity (%)	Hispanic or Latino	17 (12.8)	49 (20.9)
	Not Hispanic or Latino	116 (87.2)	185 (79.1)

Note. *SCA* Simultaneous Cannabis and Alcohol Use.

### Measures

The Alcohol Use Disorders Identification Test (AUDIT; (Saunders et al. 1993; World Health Organization, (World n.d).) was used to measure participant alcohol consumption patterns and consequences. In 2004 Kokotalio and colleagues (Kokotailo et al. 2004) reported that the AUDIT had strong internal consistency (Cronbach’s alpha = 0.89) and was a reliable tool for identifying alcohol misuse and disorders in college student populations. Sample AUDIT items include: “How many standard drinks containing alcohol do you have on a typical day when drinking?” and “Have you or someone else been injured as a result of your drinking?” Typical alcohol frequency was ascertained from the first question of the AUDIT “How often do you have a drink containing alcohol?” with response options 0 = “Never”, 1 = “Monthly or less”, 2 = “2–4 times per month”, 3 = “2–3 times per week”, and 4 = “4 or more times per week”.

The Daily Sessions, Frequency, Age of Onset, and Quantity of Cannabis Use Inventory (Cuttler and Spradlin 2017) was used to measure participant cannabis consumption, administration, and preferences. Sample items include: “Approximately how many days of the past month did you use cannabis?” with a free response answer. Participants were asked about their most recent cannabis use occasion with the item “Which of the following best captures when you last used cannabis?” with response options including: 0 = “Over a year ago”, 1 = “9–12 months ago”, 2 = “6–9 months ago”, 3 = “3–6 months ago”, 4 = “1–3 months ago”, 5 = “Less than 1 month ago”, 6 = “Last week”, 7 = “This week”, 8 = “Yesterday”, 9 = “Today”, and 10 = “I am currently high”.

SCA use frequency was examined by asking only participants who reported previous cannabis and alcohol use “How often do you use cannabis and drink alcohol at the same time? (Use one while feeling the effect from the other)”. Response options included 0 = “Less than once per month”, 1 = “One day a month”, 2 = “Two days a month”, 3 = “Three days a month”, 4 = “One day a week”, 5 = “Two days a week”, 6 = “Three days a week”, 7 = “Four days a week”, 8 = “Five days a week”, 9 = “Six days a week”, and 10 = “Daily”. Participants who indicated less than monthly SCA use were coded as a 0, while participants who indicated at least monthly SCA use were coded as a 1. Note that to receive this question, participants were not required to report SCA use, meaning some individuals in the SCA- group may have never engaged in SCA use, but all participants reported lifetime alcohol and cannabis use.

The Depression, Anxiety, and Stress Scale (DASS; Lovibond and Lovibond 1995b) was used to measure levels of depression, anxiety, and stress in all participants. The scale had high internal consistency (Cronbach’s alpha: Depression = 0.91, Anxiety = 0.84, Stress = 0.90) in previous research, suggesting that the DASS is a reliable measure for assessing negative emotional states in college populations. Sample DASS items include “I felt downhearted and blue”; “I felt I was close to panic”; “I found it difficult to relax.” DASS responses were assessed on a scale from 0 (“did not apply to me at all”) to 3 (“applied to me very much, or most of the time”), and items comprising each subscale were summed. Greater prevalence of DASS symptoms equated to higher domain scores.

### Analysis plan

All data management and initial statistical testing for random differences between groups on model variables was conducted using R statistical software (version 2023.03.0 + 386), specifically the tidyverse (Wickham et al. 2019), MplusAutomation (Hallquist and Wiley 2018), and car (Fox et al. 2024) packages. Participants were first coded into their respective groups: SCA + including only individuals who reported typically using alcohol and cannabis simultaneously at least once per month, and SCA- including individuals who reported typically using alcohol and cannabis simultaneously less than once per month. The groups were compared using t-tests on age, typical alcohol use, and most recent cannabis use occasion, as well as using chi squared tests on sex, gender, race, and ethnicity. Note that for all analyses, ‘sex’ refers to the participant’s sex assigned at birth.

Next, a multigroup path analysis was conducted using M*plus* 8 (Muthén and Muthén 1998). While the current study is cross-sectional and cannot establish temporal precedence, given previous longitudinal research indicating a positive relationship between mental health outcomes and preceding co-use behaviors (Fleming et al. 2021; Thompson et al. 2021), we elected to assess SCA as a predictor of mental health outcomes. Individual DASS symptom domains (depression, anxiety, and stress subscales) were simultaneously regressed onto the dichotomously coded SCA group variable, age, most recent cannabis use occasion and typical alcohol use frequency, with sex as the grouping variable. All predictor variables were allowed to freely covary, and all outcome variables were allowed to freely covary. Parameters of the multigroup path analysis were estimated using maximum likelihood estimation and significant results were interpreted at *p* < 0.05. To assess overall model fit, criteria suggested by Hu and Bentler (Hu and Bentler 1999) were used, including the chi-squared goodness-of-fit test, Comparative Fit Index (CFI) > 0.95, and Root Mean Square Error of Approximation (RMSEA) < 0.08. To examine whether individual paths in the model significantly differed between males and females, z-tests were conducted using model constraints (Brown 2015) to assess whether differences existed in the relations between predictors and outcomes across sex. The z-tests were conducted such that a difference score was created between males and females for the coefficients linking predictors to outcomes, and these difference scores were tested to examine whether they were significantly different from 0. Z scores that were significantly different from 0 indicated that the relation between those variables was significantly different between males and females. To examine whether the current sample provided enough power to adequately assess the research questions, post-hoc Monte Carlo simulations based on estimates from the current model were run and sample size recommendations are provided for future research.

## Results

**Fig. 1:**
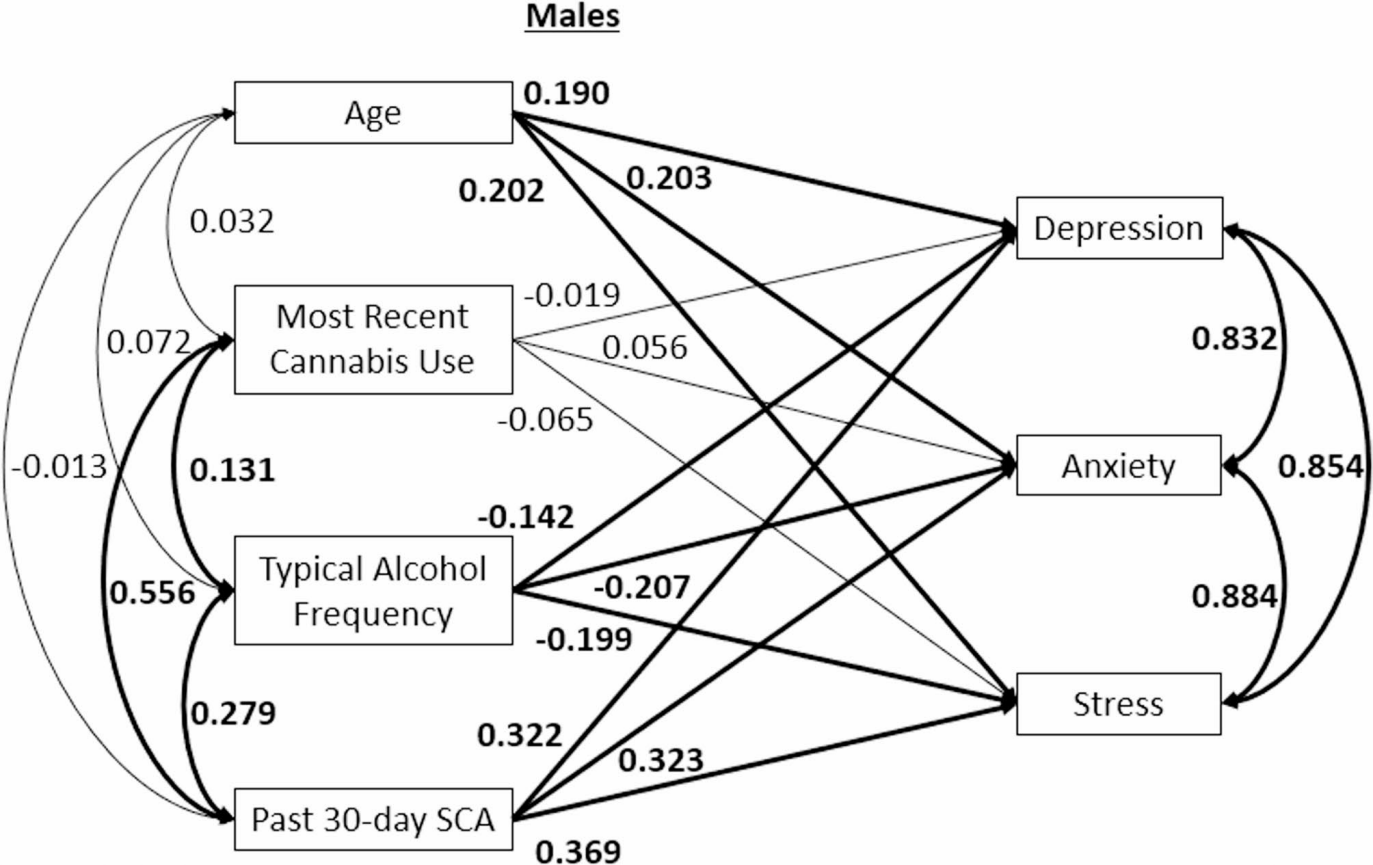
Standardized path model for individuals assigned male at birth. Note. Associations between age, substance use, and psychological distress are shown. Statistically significant paths (*p*<.05) are bolded. All variables are observed. SCA = Simultaneous Cannabis and Alcohol Use

**Fig. 2:**
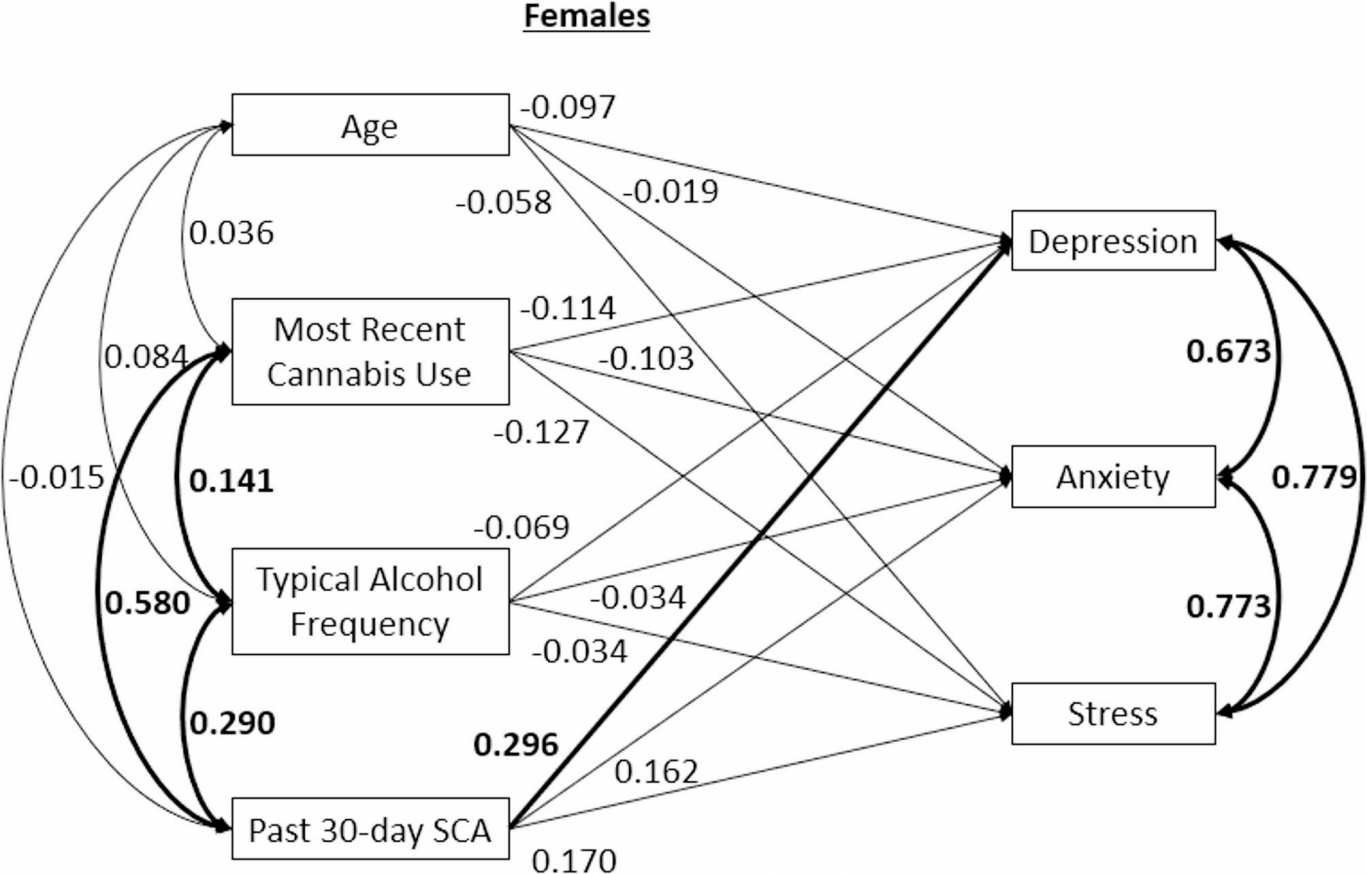
Standardized path model for individuals assigned female at birth. Note. Associations between age, substance use, and psychological distress are shown. Statistically significant paths (*p*<.05) are bolded. All variables are observed. SCA = Simultaneous Cannabis and Alcohol Use

The groups did not display significant differences on age, sex, gender, race or ethnicity. (*p*’s > 0.05). The groups did significantly differ on typical alcohol use frequency, *t*(365) = −5.81, *p* < 0.001, and most recent cannabis use occasion, *t*(363.58) = −15.79, *p* < 0.001. These variables were controlled in the analyses by including them as covariates. Because covariances among predictor variables, and among outcome variables, were constrained to be equal across sexes, a correlation table for the overall sample is provided in Table 3. The model reflected excellent fit to the data based on the chosen fit indices: chi squared test of model fit = 14.746 (*p* = 0.098), CFI = 0.992, RMSEA = 0.059. Results in the analysis for male students (see Fig. 1) indicated that the SCA + group (relative to SCA-) showed significantly higher scores on depression (β = 0.322, *p* < 0.001), anxiety (β = 0.323, *p* < 0.001) and stress (β = 0.369, *p* < 0.001). Age showed a significant positive association to scores on depression (β = 0.190, *p* = 0.024), anxiety (β = 0.203, *p* = 0.007), and stress (β = 0.202, *p* = 0.008). Typical alcohol use frequency showed a significant negative association to scores on depression (β = −0.142, *p* = 0.042), anxiety (β = −0.207, *p* = 0.001) and stress (β = −0.199, *p* = 0.001). Most recent cannabis use occasion showed no significant associations to scores on depression (β = −0.019, *p* = 0.793), anxiety (β = 0.056, *p* = 0.381), or stress (β = −0.065, *p* = 0.364). For females (see Fig. 2), SCA + group membership was associated with significantly higher scores on depression (β = 0.296, *p* = 0.001), but not anxiety (β = 0.162, *p* = 0.114) or stress (β = 0.170, *p* = 0.063). Age was not significantly associated with depression (β = −0.097, *p* = 0.069), anxiety (β = −0.019, *p* = 0.775), or stress (β = −0.058, *p* = 0.339) in female students. Typical alcohol use frequency was not significantly related to scores on depression (β = −0.069, *p* = 0.346), anxiety (β = −0.034, *p* = 0.697), or stress (β = 0.034, *p* = 0.666). Most recent cannabis use occasion did not show a significant association with depression (β = −0.114, *p* = 0.145), anxiety (β = −0.103, *p* = 0.254), or stress (β = −0.127, *p* = 0.140).

**Table 3:** Correlation table for the overall sample

Variable	1	2	3	4	5	6
1. Age						
2. Cannabis Frequency	− 0.06					
	[−0.16, 0.04]					
3. Alcohol Frequency	− 0.02	0.14**				
	[−0.12, 0.09]	[0.04, 0.24]				
4. SCA Group Membership	− 0.01	0.58**	0.29**			
	[−0.11, 0.09]	[0.51, 0.64]	[0.19, 0.38]			
5. Depression	0.10	− 0.03	− 0.01	− 0.04		
	[−0.01, 0.20]	[−0.13, 0.07]	[−0.11, 0.09]	[−0.15, 0.06]		
6. Anxiety	0.05	− 0.07	0.01	− 0.06	0.78**	
	[−0.05, 0.15]	[−0.17, 0.03]	[−0.09, 0.11]	[−0.16, 0.04]	[0.74, 0.82]	
7. Stress	0.04	− 0.05	− 0.02	− 0.02	0.75**	0.71**
	[−0.07, 0.14]	[−0.15, 0.05]	[−0.12, 0.08]	[−0.12, 0.08]	[0.70, 0.79]	[0.65, 0.76]

Note. Values in square brackets indicate the 95% confidence interval for each correlation. The confidence interval is a plausible range of population correlations that could have caused the sample correlation. * indicates *p* < 0.05. ** indicates *p* < 0.01. *SCA* Simultaneous Cannabis and Alcohol Use.

The z-tests conducted to compare coefficients across males and females yielded results showing that the relations between age and depression (B = −1.832, *p* = 0.004), anxiety (B = −0.989, *p* = 0.040), and stress (B = −1.505, *p* = 0.010) were found to be significantly larger for males compared to females. All other relations between predictor variables and outcomes displayed non-significant results (*p*’s > 0.05) when compared across sex. Estimated effects of outcomes on predictors grouped by sex, along with results from the z-tests, are shown in Table 4.

**Table 4:** Multigroup path analysis results

*Males*	*β*	*SE*	*p* value
*Outcome variable: Depression*			
Age	0.190	0.084	0.024*
Cannabis Use Frequency	−0.019	0.072	0.793
Alcohol Use Frequency	−0.142	0.070	0.042*
Monthly SCA Use	0.322	0.090	< 0.001***
*Outcome variable: Anxiety*			
Age	0.203	0.075	0.007**
Cannabis Use Frequency	0.056	0.064	0.381
Alcohol Use Frequency	−0.207	0.062	0.001**
Monthly SCA Use	0.323	0.076	< 0.001***
*Outcome variable: Stress*			
Age	0.202	0.076	0.008**
Cannabis Use Frequency	−0.065	0.072	0.364
Alcohol Use Frequency	−0.199	0.061	0.001**
Monthly SCA Use	0.369	0.089	< 0.001***

*Females*			
*Outcome variable: Depression*			
Age	−0.097	0.053	0.069
Cannabis Use Frequency	−0.114	0.078	0.145
Alcohol Use Frequency	−0.069	0.073	0.346
Monthly SCA Use	0.296	0.085	0.001**
*Outcome variable: Anxiety*			
Age	−0.019	0.065	0.775
Cannabis Use Frequency	−0.103	0.091	0.254
Alcohol Use Frequency	−0.034	0.088	0.697
Monthly SCA Use	0.162	0.103	0.114
*Outcome variable: Stress*			
Age	−0.058	0.061	0.339
Cannabis Use Frequency	−0.127	0.086	0.140
Alcohol Use Frequency	−0.034	0.079	0.666
Monthly SCA Use	0.170	0.091	0.063

*Z-Test Comparisons (Females vs. Males)*	*B*	*SE*	*p* value
SCA à Depression	−0.206	2.693	0.939
SCA à Anxiety	−2.177	1.996	0.276
SCA à Stress	−3.881	2.448	0.113
Cannabis Frequency → Depression	−0.335	0.355	0.346
Cannabis Frequency → Anxiety	−0.391	0.275	0.156
Cannabis Frequency → Stress	−0.174	0.334	0.602
Alcohol Frequency → Depression	0.742	1.245	0.551
Alcohol Frequency → Anxiety	1.399	0.991	0.158
Alcohol Frequency → Stress	1.775	1.098	0.106
Age → Depression	−1.832	0.628	0.004**
Age → Anxiety	−0.989	0.482	0.040*
Age → Stress	−1.505	0.580	0.010*

Note. Significant effects are indicated by boldface font and the following: *p* < 0.05*, *p* < 0.01**, *p* < 0.001***. Standardized effect size values are provided for all paths except in the model comparisons across sex section, where reported values are unstandardized. *SCA* Simultaneous Cannabis and Alcohol Use.

Finally, we conducted post-hoc power analyses via Monte Carlo simulations across 10,000 replications to ensure stable estimates. The Monte Carlo simulation estimates power for detecting significant differences based on the coefficients representing the strength of relations among variables obtained from the present analysis. Simulations indicated that in our sample of *N* = 367 (245 females and 122 males), we were powered to detect a difference between males and females on the relation between SCA → depression 5.4% of the time, SCA → anxiety 21.6% of the time, and SCA → stress 36.6% of the time. Additional simulations based on coefficient estimates from the current sample indicated that to detect a difference between SCA → anxiety and SCA → stress at 80% power would require a total sample size of *N* = 2050 (1025 males and females), and *N* = 1000 (500 males and females), respectively.

## Discussion

The increasing prevalence of SCA use (Cerdá et al. 2020; McCabe et al. 2021) and related consequences (Brière et al. 2011; Cummings et al. 2019; Fleming et al. 2021; Jackson et al. 2020; Linden-Carmichael et al. 2019, 2020; Patrick et al. 2022; Subbaraman and Kerr 2015; White et al. 2019) underscores the importance of studying SCA behaviors in college students. Greater knowledge of consequences related to SCA and how different variables (i.e., sex and frequency of use) impact these consequences may provide insight into potential intervention and prevention approaches for college students who co-use alcohol and cannabis and present with mental health challenges.

We found that monthly or greater SCA use is significantly associated with greater depression symptoms for both male and female college students, consistent with prior research finding that more frequent SCA use correlates with more severe mental health symptoms (Fleming et al. 2021; Patrick et al. 2022; Subbaraman and Kerr 2015; Thompson et al. 2021). We also report that SCA use is associated with higher anxiety and stress in males but not in females, however the strength of these relationships did not significantly differ between sexes. Post-hoc power analyses indicated that the present study was underpowered to detect sex differences in this sample. While larger samples are needed to clarify these associations, the observed pattern of results broadly aligns with evidence that in non-clinical samples, males may engage in heavier or more coping-driven substance use than females (Park and Levenson 2002), which may help explain why SCA could be more strongly associated with anxiety and stress in males. Adequately powered longitudinal studies should investigate these relations further. Our Monte Carlo simulations suggest sample sizes of at least 1000 are needed to detect sex differences in the association between SCA and stress at 80% power, and larger samples (*N* = 2050) are needed to detect sex differences in the relationship between SCA and anxiety. For the relationship between SCA and depression, increasing sample size did not improve power in the present study (for both a sample size of 367 and 2200, power hovered at approximately 5%), indicating a true sex difference may not exist between these variables.

Interestingly, we found that typical alcohol use frequency *negatively* predicted depression, anxiety, and stress among males but not among females. Although these sex differences also did not reach significance in the present sample, the pattern of results observed in males—negative associations between alcohol and mental health but positive associations between SCA and mental health—are notable and worth exploring in larger studies. It may be that while SCA use is associated with overall worse mental health among male college students, alcohol use on its own reflects social drinking and/or serves as a short-term stress buffer. Indeed, compared to young adult women, who are more than twice as likely to experience depression and anxiety than men (Cyranowski et al. 2000; Salk et al. 2017), and who tend to rely on emotion-focused coping strategies such as rumination and seeking social support (Tamres et al. 2002), young adult men report lower baseline internalizing symptoms and more frequent use of avoidant or externalizing coping strategies such as substance use (Park and Levenson 2002). Thus, for male college students, alcohol use may serve as an avoidant coping strategy with acute stress-reducing effects (Claus et al. 2022; Linden-Carmichael et al. 2021), whereas for female college students, who already report higher baseline internalizing symptoms and may rely more on emotion-focused coping, alcohol use may not play the same role. Future longitudinal studies with adequate power to detect sex differences may be valuable to explore patterns of alcohol and cannabis use in males and females and how these behaviors interact with sex-specific coping strategies. Gaining a more detailed picture of students’ daily substance use patterns over time would also help to disentangle the specific effects of alcohol and cannabis individually on mental health outcomes among students who co-use.

The present findings may have several possible implications for prevention and/or intervention efforts for college students. First, results add to prior evidence suggesting that SCA use may be an effective intervention target for mental health problems—particularly depression—among college students (Brière et al. 2011; Fleming et al. 2021; Patrick et al. 2022). Programs aimed at preventing depression in college students may benefit from assessing detailed patterns of substance use, including co-use behaviors (Helle et al. 2022; Montemayor et al. 2023). Such prevention and intervention efforts might be particularly relevant for male students, given the significant relations between SCA use and mental health outcomes examined in this study, though future studies with larger sample sizes are needed to better understand sex differences in the relationship between SCA and mental health.

### Limitations and future directions

This study is subject to limitations related to the survey questions we asked, sample composition, and sample size. The questions regarding SCA and alcohol use assessed typical patterns of use, while for cannabis, most recent use was assessed. The SCA question assessed typical SCA patterns, resulting in potential heterogeneity within the SCA + and SCA- groups (e.g., the SCA- group may include some individuals who have never engaged in SCA). Further, this question does not identify people who engage in concurrent alcohol and cannabis use or capture the fact that some individuals may engage in both simultaneous and concurrent co-use throughout their daily life. Future studies should include more detailed questions on simultaneous and concurrent co-use to explore these potentially important nuances.

All participants in the study were college students aged 18–25, and most identified as White non-Hispanic, thus limiting generalizability to other populations. Variations in substance use and frequency have been observed based on race and ethnicity (Chen and Jacobson 2012)– thus the underrepresentation of diverse ethnic backgrounds in this sample may bias the present results towards those of a singular ethnic demographic. Future studies on this topic should include non-college populations, and more diverse (i.e., ethnically, racially, and other marginalized identities) populations. In addition, these students reside in Colorado, which was the first state to legalize recreational cannabis. Thus individuals in Colorado may have different overall attitudes toward cannabis compared to college students living in other states, particularly those with more stringent legal policies and presumably more negative attitudes toward cannabis (Carliner et al. 2017). Findings should be replicated across different universities/geographic locations including states with different cannabis policies. Finally, as revealed by the post-hoc power analysis, the small sample sizes (N_SCA+_ = 133, N_SCA−_ = 234; 245 females and 122 males) indicate the current study was underpowered to find statistically significant sex differences, and analyses should be replicated in a larger sample. Examining mental health outcomes across various levels of SCA use (i.e., daily, weekly, monthly) and intensity (i.e., quantity consumed) is another important future direction. Additional longitudinal research is needed to better understand the directionality, temporality, and etiology of these relations (i.e., does SCA result in mental health symptoms or vice versa).

## Conclusions

This study adds to the growing evidence linking SCA to mental health concerns in college students. Mental health providers working with college students may wish to consider incorporating detailed assessments of alcohol and cannabis co-use, as SCA use may be a useful intervention target. SCA use could also be an important factor to assess in a prevention or mental health promotion context. Future studies employing larger, more representative samples, more nuanced assessments of co-use, and longitudinal designs are needed to clarify the patterns, directionality and mechanisms connecting SCA use with mental health outcomes.

## Data Availability

The datasets used and/or analyzed during the current study are available from the corresponding author on reasonable request.

## Abbreviations

SCA: Simultaneous Alcohol and Cannabis use
SCA + group: individuals that reported SCA at least once per month
SCA- group: individuals that reported SCA less than once per month
AUDIT: Alcohol Use Disorder Identification Test
DASS: Depression, Anxiety, and Stress Scale
CFI: Comparative Fit Index
RMSEA: Root Mean Square Error of Approximation
